# Comparative efficacy of abdominal tuina and mosapride citrate in functional constipation: A clinical trial

**DOI:** 10.1097/MD.0000000000043835

**Published:** 2025-09-05

**Authors:** Xiaoyu Shi, Lingyun Shi, Yukui Tian, Ning Li

**Affiliations:** a Department of Spinal Surgery, The First Affiliated Hospital of Xinjiang Medical University, Urumqi, China; b Department of Tuina, Affiliated Traditional Chinese Medicine Hospital of Xinjiang Medical University, Xinjiang Uygur Autonomous Region Academy of Traditional Chinese Medicine, Urumqi, China.

**Keywords:** abdominal tuina, functional constipation, improved quality of life, mosapride citrate, randomized controlled trial, tuina therapy

## Abstract

Functional constipation (FC) is a prevalent gastrointestinal disorder that can significantly impact patients’ quality of life. In this study, we aimed to evaluate the effectiveness and safety of “abdominal tuina” and oral mosapride citrate tablets in the treatment of FC. Ninety patients with FC were randomly assigned to receive either “abdominal tuina” treatment or oral mosapride citrate tablets. Symptom scores, quality of life assessments, physiological and biochemical indicators, and mental health parameters were monitored at various time points during the 90-day treatment period. Significant improvement in clinical symptom scores was observed in the experimental group by day 10, with sustained efficacy throughout the study. The experimental group demonstrated superior effectiveness and improvements in biochemical markers and mental health scores compared to the control group. No significant adverse reactions were reported in either group. The study findings suggest that “abdominal tuina” treatment and oral mosapride citrate tablets are equally effective in managing FC, with both interventions effectively reducing symptoms and enhancing quality of life without significant adverse effects. The maintenance of treatment effects at day 10 highlights the potential benefits of these therapies for FC patients.

## 1. Introduction

Functional constipation (FC) is a common gastrointestinal functional disorder characterized by symptoms such as difficulty in bowel movements, decreased frequency of bowel movements, and hard stool, according to the Rome diagnostic criteria.^[1–3]^ It has been estimated that FC affects approximately 14% of the global adult population, with a higher prevalence among women and the elderly.^[3,4]^ FC not only significantly impacts a patient’s quality of life but may also lead to various physical and mental health issues, such as anal fissures, hemorrhoids, anxiety, and depression.^[1–3]^ Although FC does not pose a direct threat to life, its effects on daily activities, work efficiency, and the mental health of individuals should not be overlooked. Therefore, finding effective treatment methods is essential for improving patients’ quality of life.

Current treatment methods for FC mainly include drug therapy, dietary adjustments, and lifestyle changes.^[5,6]^ Common drug treatments such as laxatives and intestinal lubricants can provide short-term relief of symptoms; however, long-term use may lead to side effects such as dependency and electrolyte imbalances.^[7]^ Additionally, some patients may exhibit poor responses to drug treatment or have difficulty tolerating the side effects of medications^[8–10]^ Lifestyle adjustments, including increased dietary fiber intake and regular physical activity, may be effective for some patients but have limited efficacy for those with severe or chronic constipation. Therefore, research and development of new treatment methods are crucial directions in the current study of FC treatment.

“Abdominal tuina,” as a traditional physical therapy method with a history of hundreds of years in traditional Chinese medicine, involves massaging specific abdominal acupoints to regulate intestinal function, improve blood circulation, and enhance the functional status of visceral organs. In recent years, with a growing interest in alternative and complementary medicine, “abdominal tuina” has gained attention as a noninvasive treatment option in the management of FC.^[11]^ Initial clinical studies have shown that “abdominal tuina” can effectively alleviate constipation symptoms, increase bowel movement frequency, and have minimal side effects. Moreover, this method is easy to learn and implement, and patients can perform it at home, making it suitable for long-term management and self-care.

This study aims to systematically evaluate the effectiveness and safety of “abdominal tuina” in the treatment of FC through a randomized controlled trial, comparing it with the current standard drug therapy (mosapride citrate tablets). By randomizing and following 90 patients with FC for up to 90 days, this study extensively records changes in patient’s clinical symptoms, quality of life, physiological and biochemical indicators, and mental health status. The scientific and clinical significance of this study lies in validating the practicality and effectiveness of “abdominal tuina” as a non-drug treatment method in clinical practice and exploring its potential physiological and psychological mechanisms. The results of this study are expected to provide more treatment options for FC, especially for patients seeking alternative and complementary treatment methods. Through the findings of this study, we hope to provide scientific evidence for the comprehensive management of patients with FC, thereby improving their treatment outcomes and quality of life.

## 2. Materials and methods

### 2.1. Study subjects

**Figure 1.**
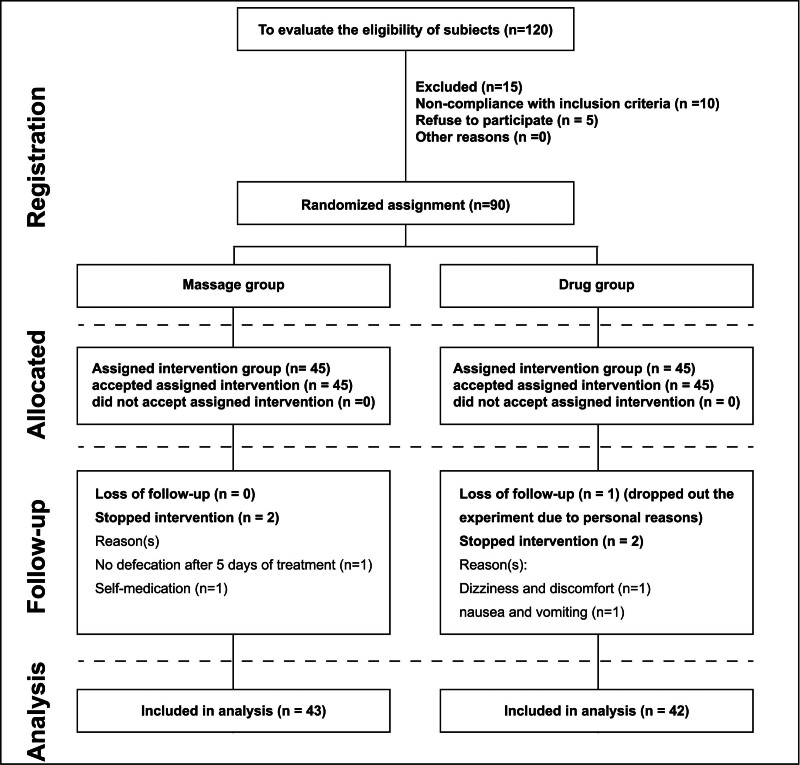
Flowchart of recruitment, retention, and allocation of participants in a randomized controlled trial.

This study is a prospective randomized controlled trial, conducted in the Department of Gastroenterology at Xinjiang Uyghur Autonomous Region Hospital of Traditional Chinese Medicine. All study subjects are patients with FC who were admitted to the Gastroenterology Department. The study process is depicted in the experimental Figure 1, which was developed based on the CONSORT guidelines.^[12]^ The trial is registered with the Chinese Clinical Trial Registry (Registration No: ChiCTR1900020744).

Based on the sample size calculation formula for high-throughput studies, the experiment requires 80 valid samples. Assuming a dropout rate of 10%, the actual minimum sample size per group is 44, and a total of at least 88 samples is needed. A total of 90 FC patients were recruited. At the onset of the trial, a total of 120 FC patients were observed; 15 patients were excluded, and 10 patients did not meet the inclusion criteria. Five patients refused to participate in the trial due to a lack of interest or suspicion. All patients were admitted for routine treatment and rehabilitation treatment in the Gastroenterology Department.

All patients in this study participated voluntarily. Participants aged between 18 and 90 years, including both male and female patients. All patients were diagnosed with FC according to the Rome IV diagnostic criteria established by the Functional Gastrointestinal International Disorders Society (FGIDS) and officially released in 2016. Patients must have experienced symptoms for at least 6 months before diagnosis and met the criteria for at least the previous 3 months.

Patients with the following symptoms were excluded: organic gastrointestinal diseases such as gastrointestinal tumors or ulcerative colitis; systemic diseases involving the gastrointestinal tract such as spinal cord injury or hypothyroidism; mechanical intestinal obstruction; constipation caused by calcium channel antagonists, anticholinergic drugs, or opioid formulations; concurrent life-threatening diseases; psychosis, depression, or anorexia nervosa; acute gastrointestinal diseases or surgical interventions within the previous month.

### 2.2. Intervention

Basic treatment: Through health education, patients are informed about the causes of constipation and further treatment options to improve patient compliance and help them adjust their lifestyles. Patients are advised to increase dietary fiber intake to 25 to 35 g/d and increase water intake to at least 1500 to 2000 mL/d; engage in moderate exercise; establish good bowel habits, such as going to the toilet regularly at the same time each day, performing a 10-minute defecation training session in the morning or 2 hours after meals, regardless of the urge to defecate, andfocusing attention on defecation and reducing external distractions.

The experimental group uses the “abdominal tuina” method for treatment, focusing on 3 acupoints: Tianshu (ST25), GuanYuan (RN4), and Zhongwan (RN12). The 3 tuina techniques employed are kneading, vibrating, and pushing on the abdomen.

Treatment principle: Regulate the internal organs and relieve constipation.

The locations and selection of the 3 abdominal acupoints are as follows: Tianshu (ST25) is located 2 inches beside the umbilicus; GuanYuan (RN4) on the midline anteriorly, 3 inches below the umbilicus; Zhongwan (RN12) on the midline anteriorly, 4 inches above the umbilicus. Both sides are simultaneously stimulated at Tianshu (ST25).

Methods and procedures involve cyclic kneading, vibrating, and pushing on the abdomen.

1. In the supine position, the doctor applies consistent pressure of 30 to 40 Newtons on the Tianshu (ST25), GuanYuan (RN4), and Zhongwan (RN12) acupoints using the finger-pushing technique for 2 to 3 minutes per acupoint. Subsequently, the doctor vibrates each of the acupoints Tianshu (ST25), GuanYuan (RN4), and Zhongwan (RN12) for 1 minute using the finger-vibrating method to induce a sensation of soreness and distension in the patient.
2. In supine position, the doctor stands or sits on the patient’s right side and performs clockwise abdominal kneading for 8 minutes with 1 hand, centering around the navel and moving outward in circles with deep pressure to the abdomen, causing the patient to feel warmth in the abdomen, thus stimulating bowel sounds and the urge to defecate.
3. In supine position, the doctor stands on the patient’s right side, clasps both hands together with fingers interlaced to form the shape of “eight,” and proceeds to push from the lower end of the rib cage in the upper abdomen to the pubic symphysis in the lower abdomen for 20 repetitions.

Treatment Course: Each treatment session is conducted 1 hour after breakfast and lunch, lasting about 20 minutes per session. A course of treatment consists of 5 sessions, with a total of 2 courses. Assessment of effectiveness is performed at the end of each course.

The control group received oral medication treatment. The control group took 5 mg of mosapride citrate tablets (manufactured by Shandong Lunan Beite Pharmaceutical Co., Ltd., Linyi City, Shandong Province, China, H19990317) 3 times a day. Each course of treatment lasted for 5 days. Two courses of treatment were administered, followed by an assessment of efficacy at the end of the treatment. During the medication treatment process, each patient completed a detailed medication usage record form, recording the specific times of taking the mosapride citrate tablets 3 times a day. In addition, patients also filled out a detailed form for medication reactions and side effects, including symptoms such as gastrointestinal discomfort, headache, dizziness, etc. Researchers verified and summarized these records weekly to ensure accuracy and consistency.

Note: During the treatment period, both groups of patients receive guidance to increase dietary fiber and water intake, maintain a light diet, adhere to good bowel habits, increase physical activity, and discontinue the use of other laxatives (if applicable).

The practitioners performing abdominal tuina in the treatment group are all senior physicians or above, totaling 3 doctors. Uniform training on techniques and acupoint localization was conducted 1 week before the study implementation. Training sessions were carried out daily for half an hour each to ensure consistency in the techniques and acupoint localization among the 3 doctors. The abdominal tuina treatment protocol and training outcomes are approved by an expert panel for this experiment.

### 2.3. Observational indicators

The constipation scoring system or Cleveland clinic score is a commonly used clinical efficacy evaluation scale in China for assessing the main symptoms of constipation, including the 6 most common symptoms experienced by constipated patients.^[13]^ Severe symptoms in long-term constipation patients can decrease satisfaction with quality of life or lead to a range of psychological issues.^[14]^ The patient assessment of constipation quality of life (PAC-QOL) questionnaire is a specialized scale for evaluating the quality of life in patients with chronic constipation. It has demonstrated good reliability and validity in different constipation patient populations.^[15,16]^

In this study, the constipation scoring system and PAC-QOL were used to assess the efficacy of the participants. To enhance the quality of the measurement indicators, evaluations of treatment effects were conducted by a senior gastroenterologist (who was blinded to the participants’ groups) before the treatment started, on the 5th, 10th, 20th, 30th, and 90th day of treatment. The experiment was terminated for participants under the following circumstances: noncompliance with the treatment regimen and prescribed methods for personal reasons; incomplete collection of observed indicators or inaccurate clinical efficacy assessments or other factors affecting efficacy judgments; urgent need for handling severe adverse events or adverse reactions.

The main scoring criteria for constipation symptoms include a series of associated symptoms such as stool characteristics, frequency of difficulty in defecation, time of defecation, frequency of bloating, and sense of incomplete evacuation. Participants are required to rate each symptom themselves. Stool characteristics are determined according to the Bristol Stool Form Scale as follows: 0 points for no bloating and incomplete evacuation; 1 point for occasional occurrence; 2 points for “sometimes”; 3 points for “often.” For defecation time, scoring is based on the frequency as follows: 0 points for once every 1 to 2 days; 1 point for every 3 days; 2 points for every 4 to 5 days; 3 points for more than once every 5 days. The higher the score (0 to 18 points), the more severe the clinical symptoms.

The PAC-QOL consists of 28 questions across 4 subscales: physiological (4 items), social/psychological (8 items), concern (11 items), and satisfaction (5 items).^[17]^ Each item is scored from 1 = no time at all/completely dissatisfied to 4 = very much so/very dissatisfied. The higher the score, the lower the participant’s satisfaction with their quality of life.

### 2.4. Psychological health assessment

To assess the psychological status of patients with FC, this study utilized the Hamilton Anxiety Scale (HAMA) (Table 1) and the Hamilton Depression Scale (HAMD) (Table 2), both of which are widely used internationally and domestically and have good reliability and validity.^[18,19]^ HAMA consists of 14 items, each scored from 0 to 4, with a total score ranging from 0 to 56. It evaluates aspects of anxiety, including physical anxiety (e.g., palpitations, difficulty breathing) and mental anxiety (e.g., tension, insomnia). HAMD comprises 17 items, each scored from 0 to 4, with a total score ranging from 0 to 68. It assesses emotional states (e.g., depression, despair) and physical symptoms (e.g., decreased appetite, sleep disturbances).

**Table 1:** Hamilton anxiety rating scale (HAMA).

	Item name	Description	Scoring criteria
1	Anxious mood	Feelings of anxiety and emotional state	0: none
			1: mild
			2: moderate
			3: severe
			4: very severe
2	Tension	Physical sensations of tension and anxiety	0: none
			1: mild
			2: moderate 3: severe
			4: very severe
3	Fears	Fear of specific objects or situations	0: none
			1: mild
			2: moderate
			3: severe
			4: very severe
4	Insomnia	Difficulty sleeping, including trouble falling asleep, waking up frequently, or early awakening	0: none
			1: mild
			2: moderate
			3: severe
			4: very severe
5	Intellectual Functioning	Impaired attention, memory, and thinking abilities	0: none
			1: mild
			2: moderate
			3: severe
			4: very severe
6	Depressed Mood	Feelings of depression and low mood	0: none
			1: mild
			2: moderate
			3: severe
			4: very severe
7	Somatic Symptoms (Muscular)	Muscle tension, aches, and pains	0: none
			1: mild 2: moderate 3: severe 4: very severe
8	Somatic symptoms (sensory)	Sensory abnormalities such as numbness and tingling	0: none
			1: mild
			2: moderate
			3: severe
			4: very severe
9	Cardiovascular symptoms	Symptoms such as palpitations and chest pain	0: none
			1: mild
			2: moderate
			3: severe
			4: very severe
10	Respiratory Symptoms	Symptoms such as rapid breathing and feelings of suffocation	0: none
			1: mild
			2: moderate
			3: severe
			4: very severe
11	Gastrointestinal Symptoms	Symptoms such as nausea, vomiting, and diarrhea	0: none
			1: mild
			2: moderate
			3: severe
			4: very severe
12	Genitourinary Symptoms	Symptoms such as frequent urination and urgency	0: none
			1: mild
			2: moderate
			3: severe
			4: very severe
13	Autonomic Symptoms	Symptoms such as sweating and dry mouth	0: none
			1: mild
			2: moderate
			3: severe
			4: very severe
14	Behavior at Interview	Behavioral manifestations such as tension and restlessness during the interview	0: none
			1: mild
			2: moderate
			3: severe
			4: very severe

**Table 2:** Hamilton depression rating scale (HAMD).

	Item name	Description	Scoring criteria
1	Depressed mood	Feelings of sadness, hopelessness, helplessness, and worthlessness	0: absent
			1: mild
			2: moderate
			3: severe
			4: very severe
2	Feelings of guilt	Self-blame, feelings of guilt, remorse, and unworthiness	0: absent
			1: mild
			2: moderate
			3: severe
			4: very severe
3	Suicide	Thoughts of death, suicidal ideation, or suicide attempts	0: absent
			1: mild
			2: moderate
			3: severe
4	Insomnia: initial	Difficulty falling asleep	0: absent
			1: mild
			2: moderate
			3: severe
5	Insomnia: middle	Difficulty maintaining sleep	0: absent
			1: mild
			2: moderate
			3: severe
6	Insomnia: late	Early morning awakening	0: absent
			1: mild
			2: moderate
			3: severe
7	Work and activities	Decrease in work, activities, and interests	0: absent
			1: mild
			2: moderate
			3: severe
8	Psychomotor retardation	Slowness of thought and speech, impaired ability to concentrate	0: absent
			1: mild
			2: moderate
			3: severe
9	Psychomotor agitation	Physical restlessness and agitation	0: absent
			1: mild
			2: moderate
			3: severe
10	Anxiety (psychological)	Apprehension, worry, dread	0: absent
			1: mild
			2: moderate
			3: severe
			4: very severe
11	Anxiety (somatic)	Physical symptoms of anxiety	0: absent
			1: mild
			2: moderate
			3: severe
			4: very severe
12	Somatic symptoms (gastrointestinal)	Digestive issues such as nausea, constipation, and loss of appetite	0: absent
			1: mild
			2: moderate
			3: severe
			4: very severe
13	Somatic symptoms (general)	General physical symptoms such as headaches, back pain, fatigue	0: absent
			1: mild
			2: moderate
			3: severe
			4: very severe
14	Genital symptoms	Sexual dysfunction and menstrual disturbances	0: absent
			1: mild
			2: moderate
			3: severe
			4: very severe
15	Hypochondriasis	Preoccupation with health and fear of illness	0: absent
			1: mild
			2: moderate
			3: severe
			4: very severe
16	Weight loss	Noticeable weight loss when not dieting	0: absent
			1: mild
			2: moderate
			3: severe
			4: very severe
17	Insight	Understanding and awareness of depressive condition	0: absent
			1: mild
			2: moderate
			3: severe
			4: very severe

Both HAMA and HAMD are evaluated by psychological factors and filled out by the subjects themselves. Participants are informed of the exact meaning of each question and decide on their own. Digestive surgeons serve as the assessors for these 2 scales. Assessments are conducted at the following time points: before the treatment starts, on the 5th, 10th, 20th, 30th, and 60th day of treatment. The scoring criteria are as follows: HAMA scores 0 to 13 are considered normal, 14 to 17 indicate mild anxiety, 18 to 24 represent moderate anxiety, and ≥25 signify severe anxiety; HAMD scores 0 to 7 are classified as normal, 8 to 16 indicate mild depression, 17 to 23 represent moderate depression, and ≥24 signify severe depression.

### 2.5. Randomization to generate allocation sequences

The data management personnel utilized SAS 9.4 to generate a 1:1 randomization table and grouped the information based on the calculated sample size.

### 2.6. Mechanism for hiding the random allocation of groups

After generating random numbers and grouping information, small notes with “abdominal tuina” or “oral medication” are placed into random opaque envelopes according to a random sequence. The cover text of the random envelopes includes the experiment name, experimenter number, researcher’s name and contact information, envelope and sequence production unit name. After screening for inclusion and exclusion criteria, all participants voluntarily participate. Randomization is carried out by the recruitment staff. Researchers open the random envelopes in the order of registration and determine the grouping of participants based on the grouping scheme in the envelope. Randomization sequence numbers and grouping information are concealed until the end of the experiment. Interventions cannot be changed during the experiment. Researchers then record the intervention assigned to the patients. The staff responsible for recruiting participants and collecting medical history do not know the group information of the subjects. During and after the intervention, outcome assessors conduct follow-up assessments. Scores for clinical symptoms of constipation and PAC-QOL are recorded in CRF forms. Data entry personnel are not involved in the experiment process. After data is input into the computer, statistical analysis is carried out by data processing personnel, who provide blinding conditions for outcome assessors, data management personnel, and data statisticians throughout the study. Only the researchers execute blinding information throughout the entire process. The random allocation sequence generated is only kept by the data researchers unless there are special circumstances (serious adverse events); the random allocation sequence and grouping information must not be disclosed in any form.

### 2.7. Concealment

This study was unable to be conducted with a blinding design. A single-blind design was implemented in which researchers and participants were aware of the intervention methods, but personnel involved in outcome assessment, data entry, and data analysis were blinded.

As this was a single-center study, different interventions may have resulted in diverse outcomes and prognoses. Due to the inability to blind participants and staff during the intervention, strict measures were taken to ensure that individuals were unaware of the participants’ allocation during the data assessment process. Blinded data collectors investigated the study outcomes. Assessors received training on the evaluation procedures. Independent evaluators who were not involved in randomization or interventions were unaware of participant allocations or specific treatment measures.

In order to uphold the quality and integrity of the trial, there were specific circumstances where the blinding had to be broken for further treatment and management of patients. Researchers documented the reasons for unblinding in the corresponding case report form, and study termination could not serve as a reason for unblinding.

### 2.8. Blood sample collection and processing

Before the start of treatment, on days 5, 10, 20, 30, and 90 of treatment, 3 mL of venous blood samples were collected from both groups of patients. Immediately after blood sample collection, the samples were centrifuged at 3000 rpm for 10 minutes to separate the serum, which was then stored at −80°C for centralized testing. Serum samples were kept at −80°C until testing, slowly thawed at 4°C and equilibrated at room temperature for 30 minutes before analysis.

Gastrointestinal hormones and inflammatory markers were measured using enzyme-linked immunosorbent assay (ELISA) kits. The determination of Motilin used an ELISA kit produced by R&D Systems (Minneapolis) (Catalog: DMTL00), while Serotonin measurements utilized an ELISA kit from IBL International (Hamburg, Germany) (Catalog: RE59121). The detection procedures were conducted according to the instructions provided with the kits, including sample addition, incubation, washing, enzyme addition, color development, and termination of the reaction, with the final optical density values read at 450 nm.

The inflammatory markers assessed included C-reactive protein (CRP) and interleukin-6 (IL-6). High-sensitivity CRP (hs-CRP) measurements were performed using an ELISA kit from Thermo Fisher Scientific (Waltham) (Catalog: EHCRPHS) for CRP, and IL-6 measurements utilized an ELISA kit from BioLegend (San Diego) (Catalog: 430501) for IL-6. The operational procedures for the inflammatory marker testing followed similar steps as the gastrointestinal hormone measurements, including sample addition, incubation, washing, enzyme addition, color development, termination of the reaction, and optical density value reading at 450 nm.

### 2.9. Statistical methods

The collected data were grouped according to the above grouping scheme and analyzed using the established database. The collected data were processed using SPSS 20.0 (IBM Corp., Armonk) statistical analysis software. Quantitative data such as age, initial defecation time, clinical efficacy scores, quality of life scores, HAMA scores, HAMD scores, and levels of physiological and biochemical indicators were expressed as mean ± standard deviation. Gender data were considered qualitative data and analyzed using variance analysis. Clinical efficacy level was treated as ordinal data and analyzed using the Wilcoxon rank-sum test. Clinical symptom scores, HAMA scores, HAMD scores, and levels of physiological and biochemical indicators were compared at 6-time points using repeated measures analysis of variance for continuous variables. Pre- and posttreatment comparisons were conducted using paired *t*-tests. Between-group comparisons were performed using independent 2-sample *t*-tests, and analysis of proportions was conducted using the chi-square test. A significance level of *P* < .05 was considered statistically significant.

### 2.10. Ethical review

This study was approved by the Ethics Committee of Xinjiang Uygur Autonomous Region Health Commission (2017XE0137-1). The Ethics Committee of Xinjiang Uyghur Autonomous Region Traditional Chinese Medicine Hospital is under the jurisdiction of the Xinjiang Autonomous Region Health and Family Planning Commission. All participants in this study voluntarily participated, met the inclusion criteria of the study, and provided signed informed consent forms.

## 3. Results

### 3.1. Subject recruitment

The changes in participants at different stages are depicted in Figure 1. All experiments were conducted at the Xinjiang Uyghur Autonomous Region Hospital of Traditional Chinese Medicine. The follow-up period ranged from July 2018 to December 2018. Ten days after the completion of treatment, a telephone follow-up was conducted with the patients. Within 6 months, 90 participants were recruited and randomly assigned to the “abdominal tuina” group (45 participants) or the Mosapride Citrate group (45 participants). Five participants withdrew during the intervention (dropout rate = 5.56%). In the “abdominal tuina” group, 2 participants dropped out of the study due to personal and family reasons. In the Mosapride Citrate group, 1 participant withdrew for personal reasons, and 2 participants withdrew due to adverse reactions. The medication compliance rate in the Mosapride Citrate group was high, with 95% of patients adhering to the prescribed medication. Common side effects included mild gastrointestinal discomfort (15%), headache (10%), and dizziness (8%), with no reports of severe adverse reactions. Ultimately, 43 participants from the “abdominal tuina” group and 42 participants from the Mosapride Citrate group completed the intervention and follow-up stages, progressing to the statistical analysis.

### 3.2. Analysis of demographic and clinical characteristics of subjects before treatment

Before conducting any randomized controlled trial, ensuring balance in baseline characteristics among participants is crucial. The aim of this study is to compare the demographic and clinical characteristics at baseline between the tuina group and the medication group to confirm the comparability of the 2 groups. Table 3 illustrates data across various dimensions, including gender, age, occupation, education level, duration of illness, daily habits (such as fluid intake and fiber intake), and more.

**Table 3:** Demographic and clinical characteristics of the subjects.

Characteristics	Sum (%)	Massage group (%)	Drug group (%)	*t*/χ^2^	*P*
Gender					
Male	36 (42.35)	15 (34.88)	21 (50.00)	1.989	.158
Female	49 (57.65)	28 (65.12)	21 (50.00)		
Age					
<30	1 (1.18)	0 (0.00)	1 (2.38)	−1.154	.252
30–45	5 (5.88)	3 (6.98)	2 (4.76)		
45–60	40 (47.06)	24 (55.81)	16 (38.10)		
60–75	30 (35.29)	12 (27.91)	18 (42.86)		
75–90	9 (10.59)	4 (9.30)	5 (11.90)		
Course of disease					
<10 yr	7 (8.24)	5 (11.63)	2 (4.76)	3.417	.181
10–20 yr	73 (85.88)	34 (79.07)	39 (92.86)		
>20 yr	5 (5.88)	4 (9.30)	1 (2.38)		
Occupation					
Retired	56 (65.88)	25 (58.14)	31 (73.81)	11.735	.163
Worker	1 (1.18)	0 (0.00)	1 (2.38)		
Office worker	13 (15.29)	10 (23.26)	3 (7.14)		
Government employee	6 (7.06)	1 (2.33)	5 (11.90)		
Freelancer	3 (3.53)	2 (4.65)	1 (2.38)		
Peasant	3 (3.53)	2 (4.65)	1 (2.38)		
Professional technicians	1 (1.18)	1 (2.33)	0 (0.00)		
Enterprise administrator	1 (1.18)	1 (2.33)	0 (0.00)		
Teacher	1 (1.18)	1 (2.33)	0 (0.00)		
Education					
Junior high or below	17 (20.00)	10 (23.26)	7 (16.67)	2.494	.287
Junior high to senior high	56 (65.88)	25 (58.14)	31 (73.81)		
Senior high or above	12 (14.12)	8 (18.60)	4 (9.52)		
Daily liquid intake					
<1500–2000 mL	23 (27.06)	15 (34.88)	8 (19.05)	2.7	.1
>1500–2000 mL	62 (72.94)	28 (65.12)	34 (80.95)		
Fiber intake					
<25–35 g/d	3 (3.53)	1 (2.33)	2 (4.76)	0.37	.543
>25–35 g/d	82 (96.47)	42 (97.67)	40 (95.24)		
Laxatives usage during the study period					
Yes	12 (14.12)	5 (11.63)	7 (16.67)	0.445	.505
No	73 (85.88)	38 (88.37)	35 (83.33)		

In terms of gender distribution, there is a balanced distribution of males and females between the 2 groups. Although there is a slightly higher proportion of males in the medication group (50.00% vs 34.88%), statistical analysis shows that this difference is not significant (*P* = .158). Regarding age distribution, both groups are mainly concentrated in the 45 to 60 and 60 to 75 age brackets, with the 55 to 60 age bracket being relatively higher in the tuina group. However, the overall age distribution difference is also not significant (*P* = .252).

In terms of occupational classification, the most common occupations in both groups are retirees, followed by office workers and government employees. While there are some proportional differences in certain occupational categories (e.g., a higher proportion of office workers in the tuina group at 23.26% compared to 7.14% in the medication group), the overall distribution of occupations is not statistically significant (*P* = .163).

For education level, the majority of participants have education levels ranging from junior high school to high school, and there is no significant difference in education levels between the 2 groups (*P* = .287). Additionally, daily fluid intake and fiber intake also did not show significant differences between the 2 groups (*P* = .1 and 0.543, respectively).

In conclusion, the participants in both groups demonstrate good consistency and balance in demographic and clinical characteristics before treatment. This provides a solid foundation for further treatment effect comparisons. Therefore, it can be concluded that the tuina group and medication group exhibit high comparability before starting treatment, providing a reliable basis for subsequent efficacy evaluations and analyses.

### 3.3. Comparison of long-term clinical efficacy between abdominal tuina and medication treatment

After ensuring good comparability of baseline characteristics between the 2 groups of participants, this study further compared the treatment effects between the tuina group and the medication group. Table 4 provides detailed records of the total number of patients in each group, the number of patients with effective treatment, and the corresponding effectiveness rates, along with the calculation of the 95% confidence intervals for each group.

**Table 4:** Comparison of efficiency between the 2 groups.

Group	Total patient number	Number of patients with effective outcomes	Effective rate	95% CI	
				Lower bound	Upper bound
Massage group	43	30	69.77%	55.47%	84.07%
Drug group	42	20	47.62%	31.87%	63.37%

**Figure 2.**
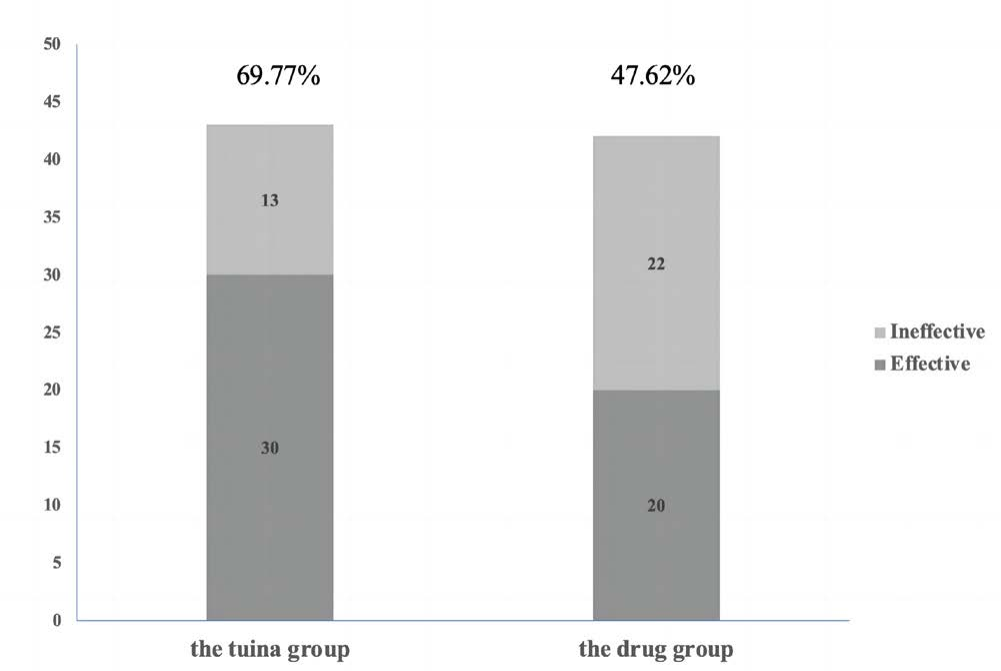
Comparison of the efficacy between “abdominal tuina” and drug treatment.

In the effectiveness assessment after treatment, the tuina group had 43 patients for final analysis, out of which 30 patients showed treatment effects, resulting in an effectiveness rate of 69.77%. The corresponding 95% confidence interval ranged from 55.47% to 84.07%, indicating a high statistical credibility for the effectiveness rate in this group. In comparison, the medication group had 42 patients, and 20 patients showed treatment effects, resulting in an effectiveness rate of 47.62%. The 95% confidence interval for this group ranged from 31.87% to 63.37%, indicating a greater variability in the treatment effects (Fig. 2).

This significant difference suggests that tuina therapy demonstrated higher effectiveness compared to medication therapy in this study. The difference in effectiveness rates is not only numerically significant, but the upper bound of the confidence interval for the tuina group is significantly higher than that of the medication group, further emphasizing the potential advantage of tuina therapy.

In summary, tuina therapy exhibited higher efficacy compared to medication therapy in this study, which may be attributed to the comprehensive effects of tuina on improving blood circulation, reducing muscle tension, and promoting body relaxation. Therefore, it can be concluded that for the condition investigated in this study, tuina therapy is a relatively more effective treatment approach.

### 3.4. Analysis of clinical symptom score changes at multiple time points for abdominal tuina and medication treatment

**Figure 3.**
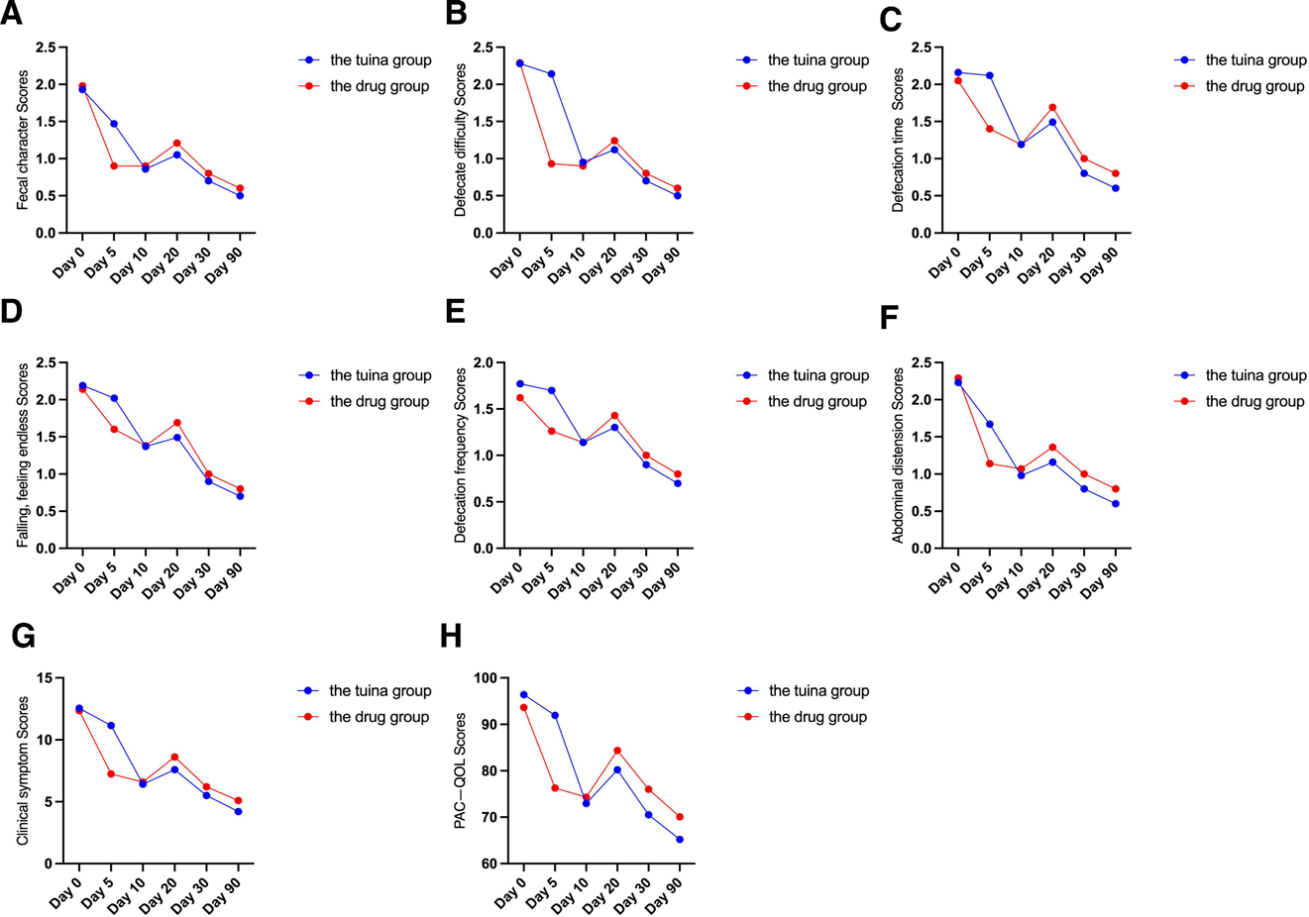
Treatment of FC: Comparison of the effects of tuina and drug interventions. (A) stool consistency; (B) difficulty in defecation; (C) defecation time; (D) feeling of incomplete evacuation; (E) number of defecations; (F) abdominal bloating; (G) total score of clinical symptoms; and (H) PAC-QOL score comparisons between the 2 groups of patients at different time points.

The repeated measures analysis of variance results shows that there are intra-group differences in fecal characteristics, difficulty in defecation, defecation time, the sensation of incomplete evacuation, frequency of bowel movements, bloating, the total score of clinical symptoms, as well as the physiological, social/psychological, satisfaction, and total scores of the PAC-QOL scale in both the treatment group and the control group (*P* < .001). This indicates that the mean values of the above symptoms and dimensions have changed over time in both groups. The inter-group effects analysis reveals significant differences (*P* < .05) between the treatment group and the control group in terms of difficulty in defecation, total score, as well as the scores in the physiological, psychological, and satisfaction dimensions of the PAC-QOL scale. There is also an interaction effect (*P* < .001) between time and treatment effects for fecal characteristics, difficulty in defecation, defecation time, sensation of incomplete evacuation, frequency of bowel movements, bloating, total score of clinical symptoms, as well as the physiological, psychological, satisfaction, and total scores of the PAC-QOL scale, indicating a significant trend of change over time with respect to these symptoms and dimensions in both groups. The results can be found in Table 5 and Figure 3.

**Table 5:** Comparison of intervention scores on days 0, 5, 10 and 20 between the tuina group and the drug group.

	The tuina group (n = 43)				The drug group (n = 42)				*F*_intra-group_	*P*_intra-group_	*F*_inter-group_	*P*_inter-group_	*F*_interaction_	*P*_interaction_
	0 d	5 d	10 d	20 d	0 d	5 d	10 d	20 d						
Clinical symptom score														
Total	12.56 ± 2.58	11.16 ± 1.70	6.42 ± 1.69	7.6 ± 2.14	12.36 ± 1.79	7.24 ± 1.81	6.60 ± 1.70	8.62 ± 1.67	194.372	<.001	7.278	.008	36.74	<.001
Fecal character	1.93 ± 0.63	1.47 ± 0.55	0.86 ± 0.64	1.05 ± 0.79	1.98 ± 0.68	0.90 ± 0.73	0.90 ± 0.73	1.21 ± 0.81	64.656	<.001	0.439	.509	8.138	<.001
Defecate difficulty	2.28 ± 0.67	2.14 ± 0.68	0.95 ± 0.72	1.12 ± 0.82	2.29 ± 0.71	0.93 ± 0.71	0.90 ± 0.66	1.24 ± 0.69	73.438	<.001	5.336	.023	23.595	<.001
Defecation time	2.16 ± 0.69	2.12 ± 0.63	1.19 ± 0.66	1.49 ± 0.74	2.05 ± 0.73	1.40 ± 0.73	1.19 ± 0.67	1.69 ± 0.64	27.400	<.001	3.784	.055	7.297	<.001
Falling, feeling endless	2.19 ± 0.73	2.02 ± 0.74	1.37 ± 0.66	1.49 ± 0.74	2.14 ± 0.68	1.60 ± 0.73	1.38 ± 0.58	1.69 ± 0.72	24.053	<.001	0.475	.492	3.701	.012
Defecation frequency	1.77 ± 0.72	1.7 ± 0.6	1.14 ± 0.64	1.3 ± 0.77	1.62 ± 0.66	1.26 ± 0.70	1.14 ± 0.68	1.43 ± 0.80	9.977	<.001	1.822	.181	2.764	.048
Abdominal distension	2.23 ± 0.61	1.67 ± 0.61	0.98 ± 0.67	1.16 ± 0.81	2.29 ± 0.51	1.14 ± 0.65	1.07 ± 0.71	1.36 ± 0.82	107.754	<.001	0.163	.688	10.027	<.001
PAC-QOL														
Total	96.40 ± 12.05	91.95 ± 13.22	72.95 ± 9.28	80.23 ± 11.29	93.64 ± 9.51	76.28 ± 7.89	74.31 ± 8.10	84.38 ± 8.80	418.361	<.001	2.419	.124	104.593	<.001
Physical	14.09 ± 3.53	13.65 ± 4.08	8.51 ± 1.79	9.35 ± 1.85	12.93 ± 3.56	8.10 ± 3.03	8.02 ± 3.12	10.38 ± 3.23	106.771	<.001	7.429	.008	44.093	<.001
Psychosocial	20.58 ± 2.00	18.74 ± 1.94	12.23 ± 1.86	15.23 ± 2.33	20.74 ± 2.18	14.00 ± 1.59	11.57 ± 1.76	17.07 ± 2.79	134.143	<.001	4.324	.041	43.238	<.001
Worries and concern	34.58 ± 8.29	33.79 ± 9.02	33.60 ± 8.67	34.67 ± 8.65	35.33 ± 8.72	34.95 ± 8.08	35.36 ± 8.28	35.48 ± 8.72	2.169	.092	0.38	.539	0.959	.408
Satisfaction	20.58 ± 2.00	18.74 ± 1.94	12.23 ± 1.86	15.19 ± 2.38	20.74 ± 2.18	14.07 ± 1.60	12.67 ± 1.78	16.10 ± 2.69	656.938	<.001	4.153	.045	97.537	<.001

### 3.5. Improvement in the psychological state of patients with FC after treatment

The results of the repeated measures analysis of variance show that the HAMA and the HAMD scores for both groups of patients have significantly decreased (*P* < .001), indicating that treatment has a sustained positive impact on the psychological well-being of patients as treatment progresses. The alleviation of constipation symptoms may reduce anxiety and depression in patients, as improvements in digestive function directly affect psychological well-being, reducing the mental burden and stress on patients.

**Figure 4.**
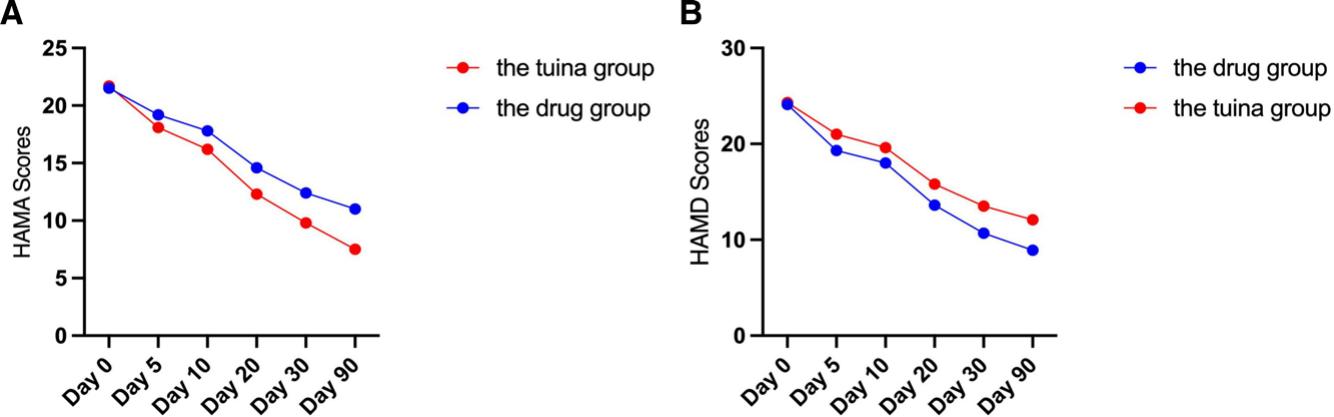
HAMA and HAMD scores of the drug group and tuina group during the study period. (A) HAMA scores; (B) HAMD scores.

The inter-group effects analysis results indicate that the HAMA and HAMD scores in the tuina group are significantly lower than those in the medication group (*P* < .05), revealing that tuina therapy is superior to medication therapy in alleviating anxiety and depression symptoms. Particularly on the 20th and 30th days after treatment, the improvement in psychological health in the tuina group is more pronounced. This may be attributed to the more direct and profound psychological soothing effects of tuina therapy through physical contact and acupoint stimulation, leading to a stronger efficacy in mental health. The results can be found in Table 6 and Figure 4.

**Table 6:** Comparison of intervention scores on days 0, 5, 10, and 20 between the tuina group and the drug group.

	The drug group (n = 42)	The tuina group (n = 43)	*F*	*P*
	Gastrin level (ng/mL)			
Day 0	110.2	109.8		
Day 5	125.4	138.7	7.45	.008
Day 10	130.6	145.9	9.32	.004
Day 20	135.8	152.1	12.57	.001
Day 30	120.5	135.6	6.89	.011
Day 90	115.2	128.3	5.76	.019

### 3.6. The effects of “abdominal tuina” therapy and medication treatment on gastrointestinal hormones and inflammatory markers

**Figure 5.**
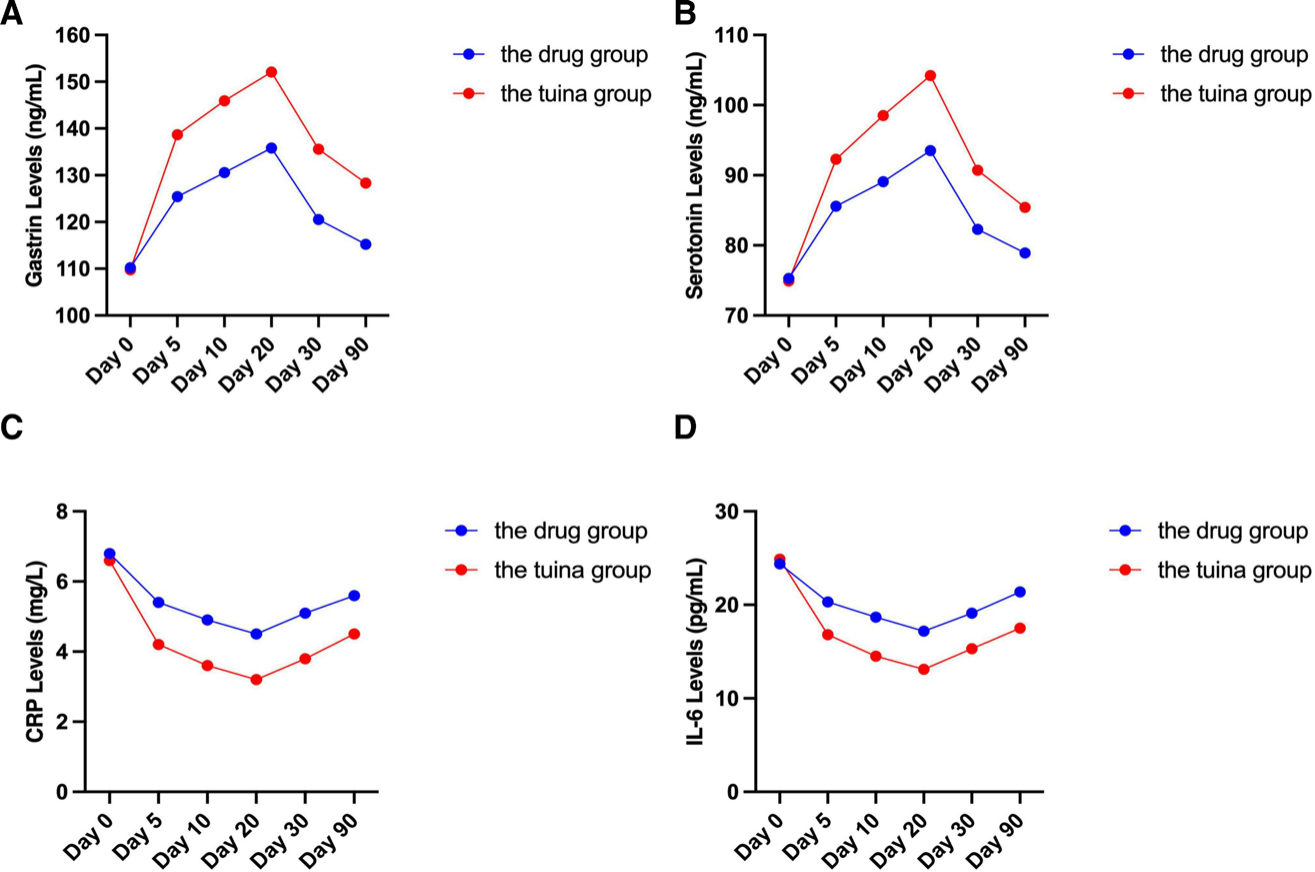
Levels of gastrointestinal hormones and inflammatory markers at different time points. Changes in the levels of gastrin, serotonin, CRP, and interleukin-6 (IL-6) at different time points in the experimental group and control group.

In this study, we measured the changes in gastrointestinal hormones (gastrin, serotonin) and inflammatory markers (CRP, interleukin-6) levels at different time points. Through repeated measures analysis of variance, the study found significant intra-group effects on gastrin, serotonin, CRP, and interleukin-6 (IL-6) levels in both the experimental group and the control group (*P* < .001), indicating significant changes in these physiological and biochemical markers over the course of the study (Fig. 5). Inter-group effects analysis further revealed significant differences (*P* < .05) between the experimental group and the control group in the changes of these markers. Additionally, the significant interaction between time and treatment effects (*P* < .001) suggests different trends in the changes of these markers at different time points for the 2 groups.

Specifically, the experimental group showed significantly higher levels of gastrin and serotonin at all time points after treatment compared to baseline levels. Gastrin levels significantly increased on the 5th, 10th, and 20th treatment days, 1-month follow-up, and 3-month follow-up (*P* < .05), and serotonin levels also markedly increased at the same time points (*P* < .05). While the control group also exhibited a significant increase in gastrin and serotonin levels at the same time points (*P* < .05), the magnitude and duration of the increase were both lower compared to the experimental group, indicating that the treatment effect was more significant and persistent in the experimental group (Fig. 5A and B).

In terms of inflammatory markers, the CRP and IL-6 levels in the experimental group significantly decreased after treatment. CRP and IL-6 levels were significantly lower than baseline levels on the 5th, 10th, and 20th treatment days, 1-month follow-up, and 3-month follow-up (*P* < .05). The control group also showed a significant decrease in CRP and IL-6 levels at the same time points (*P* < .05), but the decrease was not as significant and sustained as in the experimental group, indicating that the experimental group had a better effect in reducing inflammatory markers (Fig. 5C and D).In conclusion, “abdominal tuina” therapy not only improves intestinal function by promoting the secretion of gastrointestinal hormones but also alleviates constipation-related inflammatory responses by significantly reducing the levels of inflammatory markers. This results in significant improvements in clinical symptoms and quality of life, demonstrating superior effectiveness compared to medication therapy.

## 4. Discussion

This study evaluated the efficacy and safety of “abdominal tuina” therapy compared to traditional medication with mosapride citrate tablets for treating FC using a randomized controlled trial. The results indicate that by day 10 of treatment, patients in the experimental group demonstrated significant improvement in clinical symptoms. However, the control group showed faster improvement, and the efficacy of both groups aligned with the 10-day evaluation. Additionally, at the 20-day follow-up, the experimental group showed superior efficacy compared to the control group. This finding suggests that while “abdominal tuina” may have a slower onset of action, its long-term efficacy is comparable.

When comparing “abdominal tuina” to other non-pharmacological treatments such as biofeedback therapy and electrical stimulation therapy, the therapy displayed similar or better effects in this study. Different from other physical therapies, “abdominal tuina” is easy to perform, well-accepted by patients, and has minimal side effects. Moreover, while previous studies often focused on short-term effectiveness assessments, this study extended the observation period to 90 days, providing a more comprehensive evaluation of treatment outcomes.

In this study, patients in the experimental group showed a significant increase in levels of gastrin and serotonin, which may serve as the physiological basis for the improvement of constipation symptoms by “abdominal tuina.” Gastrin and serotonin are essential hormones in regulating intestinal motility; their elevation enhances intestinal dynamics and promotes bowel movements. This mechanism is similar to the action mechanism of some medications for constipation, but “abdominal tuina” as a non-pharmacological treatment avoids potential side effects of medications.^[20,21]^

Patients with FC often experience symptoms of anxiety and depression. The results of this study showed significant improvements in both Hamilton Anxiety Scale and Hamilton Depression Scale scores in the experimental group. This improvement not only enhances patients’ quality of life but may also be indirectly related to the improvement of intestinal function by “abdominal tuina.” Better mental health may further facilitate the normalization of gastrointestinal function, forming a beneficial cycle.

Regarding long-term effects, the study demonstrated that the efficacy of “abdominal tuina” was not significantly different from drug treatment after 10 days, and it showed superior efficacy at 20 days. This indicates its sustained efficacy and good patient compliance. Since “abdominal tuina” is a noninvasive treatment method, patients generally express willingness to undergo this type of treatment in the long term.

While providing valuable insights, this study has some limitations. Firstly, the sample size is relatively small, which may affect the generalizability of the results. Secondly, the study was conducted at a single center, possibly leading to selection bias. Lastly, not all possible confounding factors were completely eliminated, which may affect the accuracy of the results.

In conclusion, this study confirms the effectiveness and safety of “abdominal tuina” in treating FC, providing scientific evidence for non-pharmacological treatment. This finding holds significant clinical value, especially for patients who do not respond well to traditional medication or seek alternative treatment methods. Future research should expand the sample size and conduct multicenter trials to validate the results of this study’s universality. Additionally, exploring the impact of different “abdominal tuina” techniques on treatment outcomes can optimize treatment plans and maximize patient quality of life and treatment satisfaction.

## 5. Conclusion

**Figure 6.**
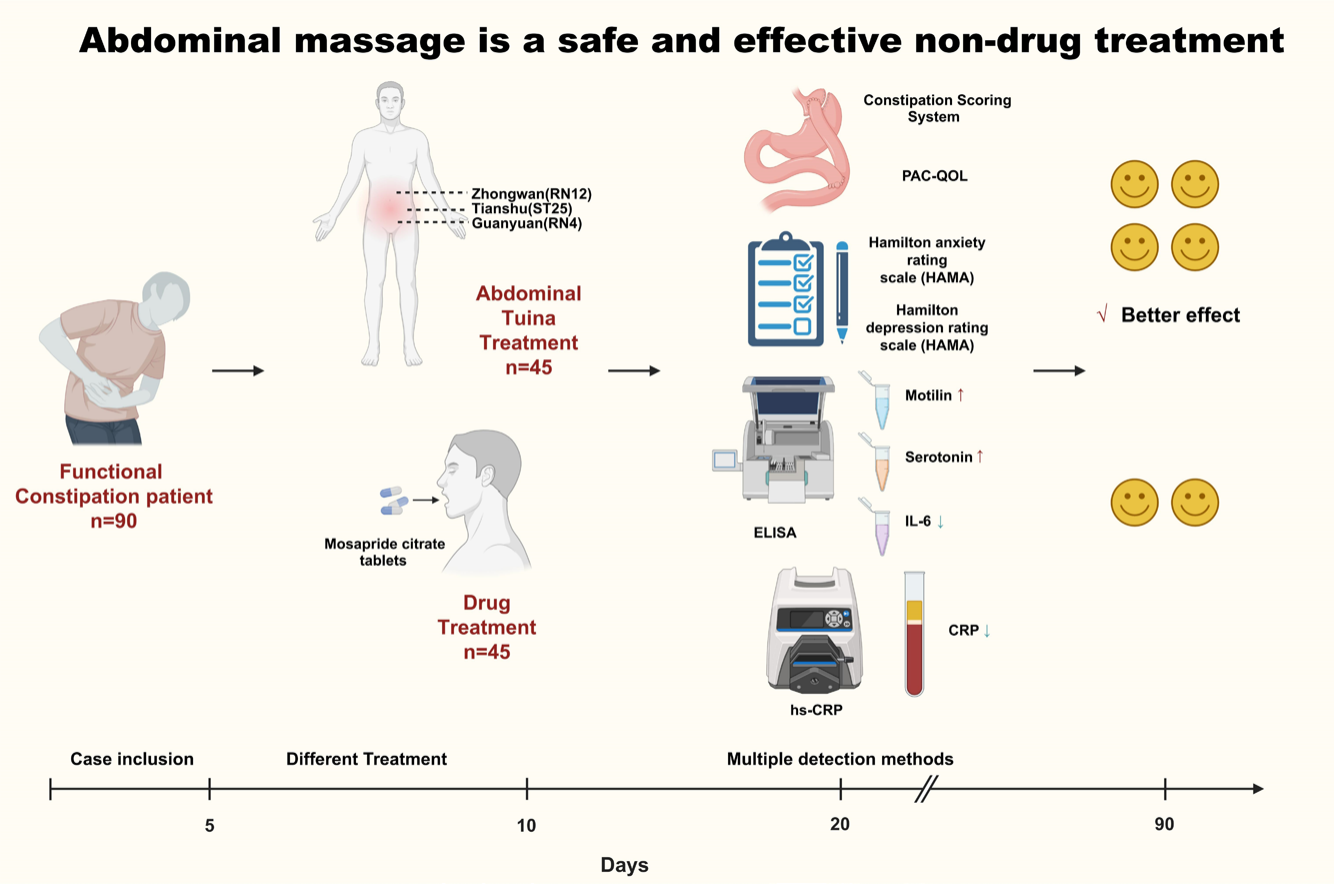
Comparison of the effects of abdominal tuina and oral mosapride citrate on the relief of symptoms of FC.

The study demonstrates that following 10 days of treatment, “abdominal tuina” therapy for FC is as effective as oral mosapride citrate medication in the control group. Both interventions significantly improved clinical symptoms with no observed side effects or adverse reactions, supporting their safety and effectiveness. At the 20-day mark, the tuina group’s effectiveness surpassed that of the drug group, indicating the sustained efficacy of tuina therapy and offering patients a non-pharmacological treatment option to enhance their quality of life (Fig. 6).

This research makes a significant contribution to the field of non-pharmacological treatment for FC, a common gastrointestinal disease. By comparing traditional tuina techniques with standard drug treatments, the study highlights the potential of comprehensive treatment approaches in managing functional disorders. The findings reveal that tuina therapy can be as effective as drug treatment, providing valuable treatment options for patients who prefer non-pharmacological interventions or may experience adverse drug reactions. Additionally, the improvement in PAC-QOL scores underscores the positive impact on patients’ quality of life, which is a crucial aspect of clinical outcomes.

“Abdominal tuina” therapy has a significant effect on alleviating constipation symptoms and improving quality of life. This finding is further validated through increased measures of physiological and biochemical indicators. Specifically, patients in the experimental group showed significantly elevated levels of gastrin and serotonin while levels of inflammatory markers decreased. These data suggest that “abdominal tuina” therapy not only effectively alleviates constipation symptoms but may also improve patients’ physiological mechanisms by regulating intestinal hormones and reducing inflammation.

The mechanisms of action of “abdominal tuina” therapy mainly involve several aspects. Firstly, by applying pressure and tuina to specific acupoints such as Tianshu (ST25), GuanYuan (RN4), and Zhongwan (RN12), the therapy stimulates the enteric nervous system, promoting gastrointestinal motility. These acupoints are closely related to gastrointestinal function, and pressure on them can effectively regulate gastrointestinal motor function, thus facilitating bowel movements. Secondly, the tuina technique improves local blood circulation, increases blood flow in the gastrointestinal tract, and enhances gastrointestinal function. Moreover, mechanical stimulation during the tuina process can regulate the endocrine system through neural reflex pathways, increasing the secretion of gastrin and serotonin, which play crucial roles in regulating gastrointestinal motility and the secretion of digestive fluids.

In addition to promoting gastrointestinal motility and regulating hormone secretion, “abdominal tuina” therapy also exhibits significant anti-inflammatory effects. The study findings indicate a significant decrease in levels of inflammatory markers in patients in the experimental group, suggesting that tuina therapy may improve constipation symptoms by reducing local intestinal inflammation. Through mechanical stimulation and reflex actions, tuina therapy may inhibit the release of inflammatory mediators, reducing intestinal mucosal inflammation and thereby aiding in alleviating constipation symptoms. Furthermore, tuina therapy can balance the autonomic nervous system, decrease sympathetic nerve activity, increase parasympathetic nerve activity, and promote gastrointestinal motility and secretion of digestive fluids.

The results of the psychological health assessment further demonstrate the unique advantages of “abdominal tuina” therapy. Patients in the experimental group showed significant reductions in scores on the HAMA and HAMD, indicating that this tuina therapy not only relieves constipation symptoms but also effectively alleviates patient anxiety and depression. This finding suggests that “abdominal tuina” therapy may indirectly improve constipation symptoms by regulating the nervous system to alleviate mental stress. In contrast, changes in the psychological health assessment in the control group were not significant, indicating limited effectiveness of mosapride citrate tablets in improving mental health. This further suggests the potential psychological advantages of “abdominal tuina” therapy in the comprehensive treatment of FC.

Long-term follow-up data validate the lasting efficacy of “abdominal tuina” therapy. Results from the extended 3-month follow-up show that the experimental group maintained good symptom relief even after the follow-up period, while some patients in the control group experienced symptom relapse. This indicates that “abdominal tuina” therapy not only has short-term efficacy but also provides lasting symptom relief. Its lasting efficacy may be attributed to its effects on patients’ physiological and psychological states through various mechanisms, including regulating intestinal hormones, reducing inflammation, and relieving mental stress. The symptom relapse observed in some patients in the control group during the follow-up period suggests that the long-term effectiveness of mosapride citrate tablets may not meet expectations, as its principal mechanisms may limit its sustained efficacy.

While the study results are promising, there are several limitations. Although the sample size of 90 patients is sufficient for initial findings, it may be relatively small for generalizing the results to a broader population. The study duration with a follow-up of only 20 days may not fully reflect the long-term effects and sustainability of tuina therapy as a treatment choice. Additionally, the study did not explore the specific mechanisms by which tuina affects intestinal motility, which could be valuable for customizing treatment based on individual patient needs.

Looking to the future, further research with larger sample sizes and longer follow-up periods is needed to validate and expand on these findings. Exploring the mechanisms by which tuina therapy impacts gastrointestinal function could provide deeper insights into its therapeutic potential. Additionally, comparing this tuina technique with other non-pharmacological treatments can establish a more comprehensive understanding of the most effective strategies for managing FC. This could lead to more personalized and effective treatment approaches, ultimately improving patient outcomes in clinical practice.

## Author contributions

**Conceptualization:** Xiaoyu Shi, Ning Li.

**Data curation:** Xiaoyu Shi, Lingyun Shi, Yukui Tian.

**Formal analysis:** Xiaoyu Shi, Lingyun Shi, Yukui Tian.

**Funding acquisition:** Ning Li.

**Investigation:** Xiaoyu Shi, Lingyun Shi, Yukui Tian.

**Methodology:** Xiaoyu Shi, Yukui Tian, Ning Li.

**Project administration:** Ning Li.

**Software:** Xiaoyu Shi, Lingyun Shi, Yukui Tian.

**Supervision:** Ning Li.

**Validation:** Lingyun Shi, Yukui Tian, Ning Li.

**Visualization:** Xiaoyu Shi, Lingyun Shi, Ning Li.

**Writing – original draft:** Xiaoyu Shi.

**Writing – review & editing:** Yukui Tian, Ning Li.

## Correction

Lingyun Shi was mistakenly listed as the corresponding author on this article when it originally published. The corresponding author has now been updated in the published article to Ning Li, with the following correspondence information: Department of Spinal Surgery, The First Affiliated Hospital of Xinjiang Medical University, No. 137, Liyuushan South Road, New Downtown, Urumqi 830000, Xinjiang Uygur Autonomous Region, China (e-mail: yfylining@163.com).
